# Arsenic in drinking water, hair, and prevalence of arsenicosis in Perak, Malaysia

**DOI:** 10.3389/fpubh.2023.998511

**Published:** 2023-02-16

**Authors:** Nurfatehar Ramly, Husna Maizura Ahmad Mahir, Wan Nurul Farah Wan Azmi, Zailina Hashim, Jamal Hisham Hashim, Rafiza Shaharudin

**Affiliations:** ^1^Environmental Health Research Centre, Institute for Medical Research, National Institutes of Health, Ministry of Health, Setia Alam, Malaysia; ^2^Department of Community Health, Faculty of Medicine, Universiti Kebangsaan Malaysia Medical Centre, Kuala Lumpur, Malaysia; ^3^Communicable Disease Control Section, Public Health Division, Perak State Health Department, Ministry of Health, Ipoh, Perak, Malaysia; ^4^Department of Environmental and Occupational Health, Faculty of Medicine and Health Sciences, Universiti Putra Malaysia, Serdang, Selangor, Malaysia; ^5^Department of Health Sciences, Faculty of Engineering and Life Sciences, Universiti Selangor, Bestari Jaya, Selangor, Malaysia

**Keywords:** arsenic poisoning, hair, water, prevalence, carcinogens, mass spectrometry

## Abstract

Arsenic is a carcinogen element that occurs naturally in our environment. Humans can be exposed to arsenic through ingestion, inhalation, and dermal absorption. However, the most significant exposure pathway is *via* oral ingestion. Therefore, a comparative cross-sectional study was conducted to determine the local arsenic concentration in drinking water and hair. Then, the prevalence of arsenicosis was evaluated to assess the presence of the disease in the community. The study was conducted in two villages, namely Village AG and Village P, in Perak, Malaysia. Socio-demographic data, water consumption patterns, medical history, and signs and symptoms of arsenic poisoning were obtained using questionnaires. In addition, physical examinations by medical doctors were performed to confirm the signs reported by the respondents. A total of 395 drinking water samples and 639 hair samples were collected from both villages. The samples were analyzed using Inductively Coupled Plasma-Mass Spectrometry (ICP-MS) to determine arsenic concentration. The results showed that 41% of water samples from Village AG contained arsenic concentrations of more than 0.01 mg/L. In contrast, none of the water samples from Village P exceeded this level. Whilst, for hair samples, 85 (13.5%) of total respondents had arsenic levels above 1 μg/g. A total of 18 respondents in Village AG had at least one sign of arsenicosis and hair arsenic levels of more than 1 μg/g. Factors significantly associated with increased arsenic levels in hair were female, increasing age, living in Village AG and smoking. The prevalence of arsenicosis in the exposed village indicates chronic arsenic exposure, and immediate mitigation action needs to be taken to ensure the wellbeing of the residents in the exposed village.

## Introduction

Arsenic (As) is a bright silver-gray metalloid which is naturally found in the Earth's crust (1, 2). Humans may be exposed to arsenic when the Earth's crust is naturally disturbed, such as through volcanic eruptions and earthquakes, or through industrial activities such as mining, burning of coal at plants and metal smelting activities (3–5). Exposure to high concentrations of arsenic can be toxic and is a public health concern. Most cases of human arsenic-induced toxicity are due to inorganic arsenic exposure. The principal route of exposure to arsenic for the general population is usually through the oral route, primarily in foods and drinking water. Studies have shown that long-term exposure to arsenic in drinking water produces a broad array of health effects (6). Prolonged ingestion (not <6 months) of arsenic above a safe dose will lead to chronic health problems identified as arsenicosis. Arsenicosis is usually exhibited by skin lesions characteristics such as hyperkeratosis, hypomelanosis, and hyperpigmentation (2). Chronic exposure to arsenic is also causally related to a severe peripheral vascular disease known as Blackfoot (7). Arsenic exposure also can affect systemic manifestations such as respiratory, hepatobiliary, cardiovascular, central nervous, gastrointestinal, hematopoietic and endocrine systems (8, 9). According to the International Agency for Research on Cancer (IARC) and US Environmental Protection Agency (USEPA), inorganic arsenic is classified as a carcinogen to human beings (10, 11). Therefore, exposed individuals have increased risks of cancers of the skin, lungs, bladder and kidney (12–14).

In Malaysia, the water supply for water usage primarily originates from rivers, and the population mainly consumes drinking water from tap water, bottled drinking water and bottled mineral water (15, 16). The primary water source in the Perak state of Malaysia is the Perak River. The Perak River is the longest in Perak and the second longest river in Peninsular Malaysia after the Pahang River. It originates from the mountains at the Perak-Kelantan-Thailand border. However, it needs to be treated in water treatment plants (WTP) before it is made potable for the residents. There are several WTP along this river, including the AG WTP. The AG WTP is located in the upper part of the Perak River and can produce 0.22 million liters per day (MLD) drinking water (17). The WTP is one of the oldest WTP and was built in 1976 at the same time as Village AG. The residents here were resettled from Village T, which was inundated when the hydroelectric dam was built in the 1970s. This AG WTP supplies treated water only to residents of AG Village. The Drinking Water Quality Program under the Engineering Division Ministry of Health is responsible for monitoring raw and treated water quality at the WTPs to ensure the consumers receive treated water free from contaminants. However, based on the monitoring data from the previous few years, there has been an occasional violation of the arsenic level in the water from the AG WTP. The presence of arsenic in treated water from the WTP has raised concerns about the potential health impact on the exposed community. Thus, the Ministry of Health and other related departments, such as the Chemistry Department, Department of Environment, Perak Forestry Department, and Perak Water Board, formed a Special Sanitary Survey. The survey aimed to identify the possible sources of arsenic contamination in raw water. Based on the survey, they found several potential activities that could contribute to arsenic in the water, affecting its quality, including mining, timber processing and agriculture activities along the river. Therefore, further evaluation and investigation must be conducted to formulate a solution to this problem. Given that there is no information and monitoring on the level of arsenic at the consumer end, hence, this study was conducted with the aims of (1) to determine the arsenic concentrations in drinking water in Village AG's households and Village P's households, (2) to determine the arsenic concentration in the hair of residents in Village AG and Village P, and lastly, (3) to determine the prevalence of arsenicosis in Village AG and Village P.

In this study, a comparative cross-sectional study was conducted to compare arsenic levels and arsenicosis prevalence between Village AG and Village P. Village AG received water supply from the WTP with high arsenic levels, whereas Village P received from the WTP with low levels of arsenic. The study involved sample collection of drinking water and hair, health surveys and physical examinations. A total of 639 participants (324 from Village AG and 315 from Village P) from 395 households (198 from Village AG and 197 from Village P) were recruited in this study. Then, the arsenic concentrations in drinking water and hair were determined and compared for both villages. The factors that increase the risk of arsenic concentration in hair have also been identified. After that, the prevalence signs and symptoms of arsenicosis were evaluated for both villages. The outcomes of this study offer baseline information for authorities to enhance water quality and ensure the populace's wellbeing.

## Materials and methods

### Study design and study area

**Figure 1:**
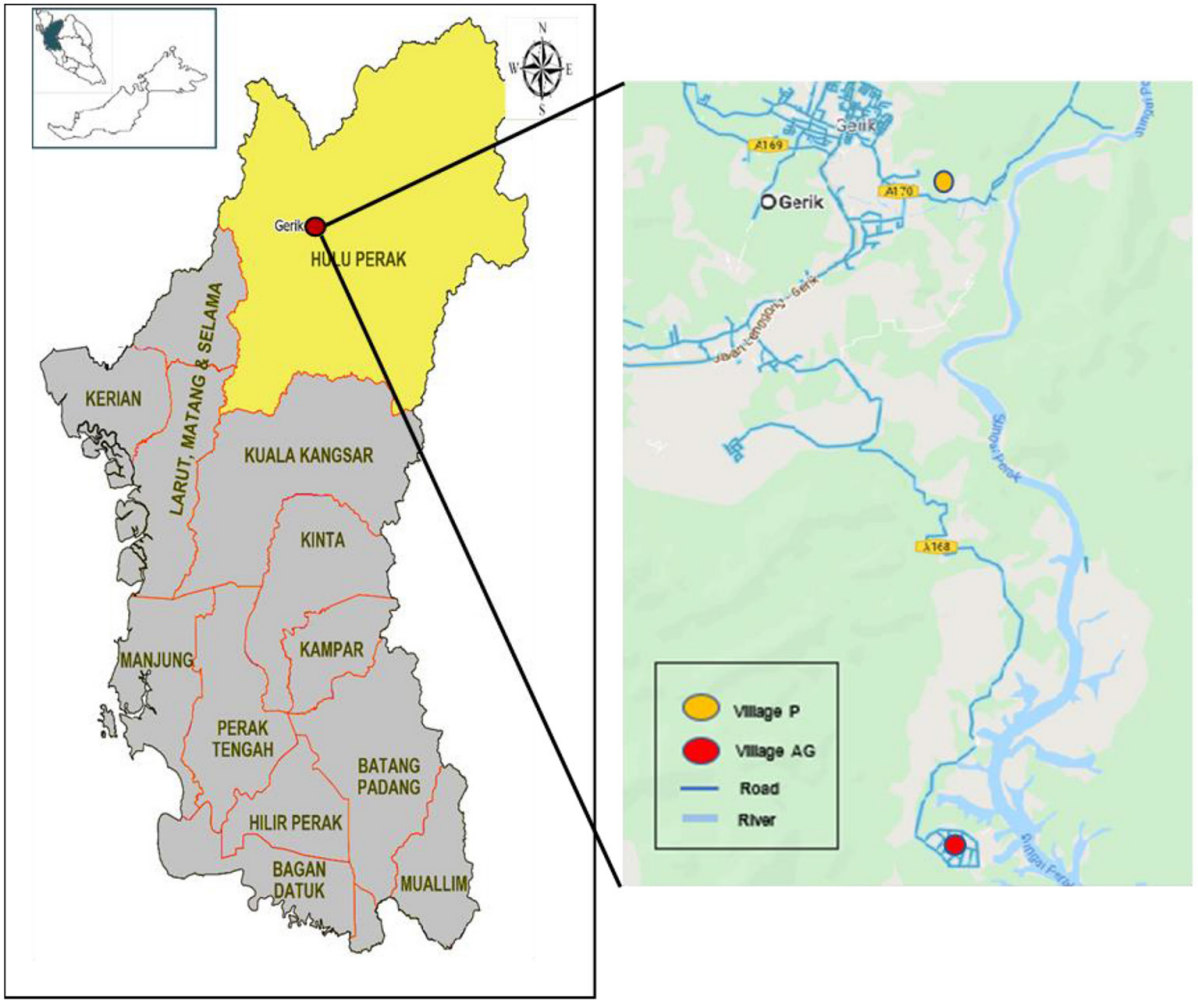
Map of Gerik in the State of Perak, Malaysia, where village AG and village P are located.

This study was conducted in Gerik (5.4168°N, 101.1164°E), one of the districts in the state of Perak, Malaysia (Figure 1). The data collection was carried out from September 2018 to October 2019. A comparative cross-sectional study design was utilized in this study to compare arsenic levels between the exposed and non-exposed groups. The exposed group were from Village AG, which received treated water from a WTP with reported violations of arsenic level. The non-exposed group were from Village P, located about 2 km away from Village AG and received treated water from a different WTP with no violations of arsenic level.

The Power and Sample Size Program software (PS version 3.0.1) was used to calculate the sufficient sample size for the cross-sectional study by comparing two proportions. For calculation, we used a power of the study of 80%, with an alpha level of 0.05 at 95% confidence level and also taking into account non-response, we inflated the sample size by 20%. Based on the calculation, 311 respondents are needed from each village to achieve a sufficient sample size. The census conducted by the Hulu Perak District Health Office showed that Village AG has 1,152 residents living in 288 houses, while Village P has about 595 residents in 132 houses. Based on the total number of residents, we estimated the average number of adults per house in Village AG and Village P to be 2.3 and 2.8, respectively. Therefore, to achieve 311 respondents from each village, a total of 135 houses were selected from Village AG and 120 houses from Village P. The study participants were selected from a universal sample of adults aged 18 and above residing in the study village for at least 1 year. Participation was voluntary, and only those given written consent were selected as study subjects. For the sampling of households, we used a random systematic sampling technique. For each village, houses were assigned a number, and SPSS software was used to randomly select the required number of houses in each village for this study.

### Sample collection, preparation, and analysis

#### Tap water samples

Before collecting water samples, the taps were turned on for 5 min to flush the water from the piping. Then, 1 L of water was collected directly into the pre-washed polyethylene bottles [pre-washed with 1% nitric acid (HNO_3_) to eliminate contaminants]. 3 ml of concentrated HNO_3_ (65%) was added to each water sample to ensure the pH was kept below 2. All the collected samples were transported and stored at 4°C. The analysis of arsenic concentration in tap water was performed according to USEPA Method 6020A using Inductively Coupled Plasma-Mass Spectrometry (ICP-MS), ELAN 9000, Perkin Elmer. About 10 ml of water was aliquoted and filtered using a 0.45 mm PTFE membrane to remove the sediments. Then, 0.3 ml HNO_3_ 65% was added to ensure the concentration of aliquots met the standard calibration curve recommended by ICP-MS analytical technique. Dilution was done with 2% HNO_3_ by preparing 18.2 Ω milliQ Ultra-Pure Water (UPW) with 65% HNO_3_. The calibration curve for tap water was prepared using standard concentrations of 2, 5, 10, 20, and 50 ppb, prepared from stock solution with 2% nitric HNO_3_ as diluent.

#### Hair samples

About 1 g of hair sample was collected from each respondent. Samples were taken at the posterior vertex and nape of the head as close as possible to the scalp using stainless steel scissors (18). Samples were sealed in labeled zipped plastic bags before being transported to the laboratory. Before analysis using ICP-MS, the hair sample was cut into small pieces at least 3 mm in length. Then, samples were washed once with 25 ml acetone, three times with 25 ml deionized water, and lastly with 25 ml acetone (19). Each washing step requires 10 min of shaking using a sonicator. After washing, the samples were dried overnight at 60°C.

Hair samples were digested using Milestone Microwave Laboratory System, ETHOS, with a rotor for 15 Teflon vessels (SK-15 high-pressure rotor). Approximately 0.1 g of dry hair samples were weighed in a clean digestion vessel. Then, 10 ml of HNO_3_ 65% Suprapur (MERCK, Germany) was added. The vessels were closed, tightened and put inside the microwave digestion system. After digestion, the vessels were taken out from the machine and opened carefully inside a fume hood after the system's temperature reached <60°C. The solutions were stored in centrifuge tubes and were diluted up to 50 ml for further analysis.

The calibration curve for hair samples analysis was prepared using standard concentrations of 5, 10, 20, 50, and 100 ppb. Sample blanks were prepared for quality control of every digestion batch to correct the background sample reading from contamination in reagents, filters or ultrapure water. The calibration check standard was analyzed at the beginning and end of the analysis and for every 10 samples for quality control purposes. The analytical method was validated using European Reference Material (ERM) DB001-human hair (Trace Element).

#### Health questionnaire and physical examination

Participation in this study was voluntary, and only those who gave written consent were selected as study subjects. Inclusion criteria were respondents aged 18 years and older who have lived in the area for at least 1 year. For each selected village, a medical team consisting of a medical doctor and a nurse or medical assistant were recruited to interview respondents. The interview was conducted using a standardized questionnaire, and a physical examination was also performed to look for signs of arsenicosis (hyperkeratosis, hypomelanosis, hyperpigmentation, and Mees' line). All team members received training related to the study from a family medicine specialist and a public health specialist. An album of images was provided to each team to help them identify the signs of arsenicosis on the skin and nails. They were also trained to collect hair samples properly.

### Statistical analysis

Statistical analysis was performed using SPSS version 26.0 for Windows (SPSS Inc., Chicago, IL, USA). First, descriptive statistics were calculated to explore and describe the data. Associations between categorical data for signs and symptoms with hair arsenic concentration were then assessed using the chi-square test. Lastly, univariate and multivariate logistic regression analyses were conducted to determine the effect of each risk factor (age, gender, duration of stay, smoking status, study villages and arsenic level in water) on the risk of having high hair arsenic level (more than 1 μg/g) among the respondents. All statistical tests were conducted at a 95% confidence interval, using *p* = 0.05.

## Results

### Descriptive analysis

A total of 639 adult respondents from Village AG and Village P consented to participate in this study. The participants included 324 respondents from Village AG and 315 respondents from Village P. Majority of respondents were females in both villages. More than 40% of respondents in both villages were housewives, students, and pensioners. The respondents were between 18 and 93 years old, with a mean age of 45.9 (SD ±14.8) years for Village AG and 46.1 (SD ±16.6) years for Village P. About 254 (78.4%) respondents in Village AG and 186 (59%) of Village P respondents lived there for more than 10 years. The data also showed that in both villages, the respondents were mostly non-smokers (Table 1).

**Table 1:** Descriptive analysis of respondents' characteristics.

**Variable**	**Village AG** **(*****n*** = **324)**		**Village P** **(*****n*** = **315)**	
	***n***	**%**	***n***	**%**
**Gender**				
Male	114	35.2	87	27.6
Female	210	64.8	228	72.4
**Age**				
Min	18	–	18	–
Max	82	–	93	–
Mean	45.9	–	46.1	–
SD	14.8	–	16.6	–
**Occupation**				
Government sector	11	3.4	113	35.9
Private sector	10	3.1	15	4.8
Self employed	133	41.0	41	13.0
Unemployed	29	9.0	15	4.8
Others^*^	141	43.5	131	41.6
**Duration of stay**				
<10 years	70	21.6	129	41
10 years and more	254	78.4	186	59
**Smoking status**				
Smoker	77	23.8	31	9.8
Non-smoker	247	76.2	284	90.1

* Others: student, housewife and pensioner.

### Arsenic concentration in drinking water and hair

The results of arsenic concentrations in drinking water and hair were summarized in Table 2. The total number of drinking water samples collected from households in Village AG and Village P was 198 and 197, respectively. The results showed that the sample collected from Village AG had the highest arsenic concentration in drinking water (0.0223 mg/L) compared to Village P (0.0017 mg/L). The hair arsenic concentrations among respondents in Village AG ranged from 0.05 to 4.15 μg/g, with a median of 0.65 μg/g. Meanwhile, hair arsenic concentration among respondents from Village P ranged between 0.04 and 1.99 μg/g, with a median of 0.17 μg/g, which was lower than respondents from Village AG.

**Table 2:** Arsenic levels in drinking water and hair by villages.

	**Drinking water** **(mg/L)**		**Hair** **(μg/g)**	
	**Village AG**	**Village P**	**Village AG**	**Village P**
*n*	198	197	324	315
Median	0.009	0.0009	0.65	0.17
Min	0.0009	0.0002	0.05	0.04
Max	0.0223	0.0017	4.15	1.99

**Figure 2:**
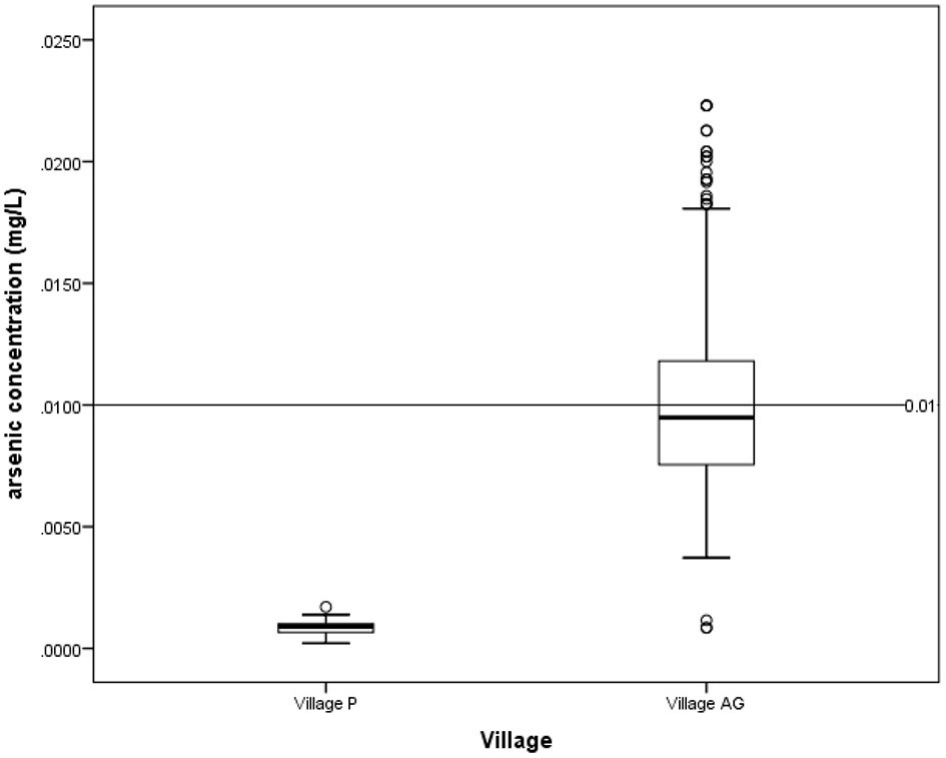
Arsenic concentration in drinking water for village P and village AG based on NDWQS guideline of 0.01 mg/L.

**Figure 3:**
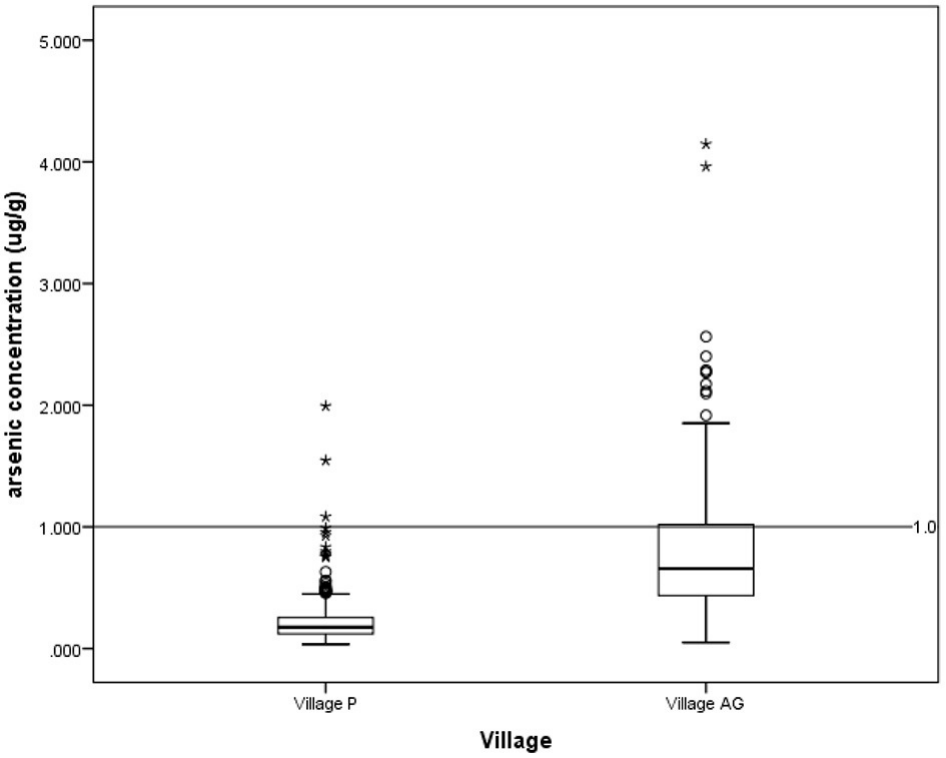
Arsenic concentration in hair for village P and village AG based on a guideline of 1.0 μg/g. *outlier.

According to the National Drinking Water Quality Standards (NDWQS) (22), water samples from 81 (41.3%) households in Village AG exceeded the maximum acceptable value of 0.01 mg/L. In contrast, none of the water samples from Village P exceeded this level (Figure 2). In contexts of hair arsenic concentration, the results showed that 82 (25%) of 324 respondents from Village AG and 3 (1%) out of 315 respondents from Village P had levels above 1 μg/g, indicating unsafe arsenic exposure (Figure 3) (20, 21).

### Prevalence of the signs and symptoms of arsenicosis

**Figure 4:**
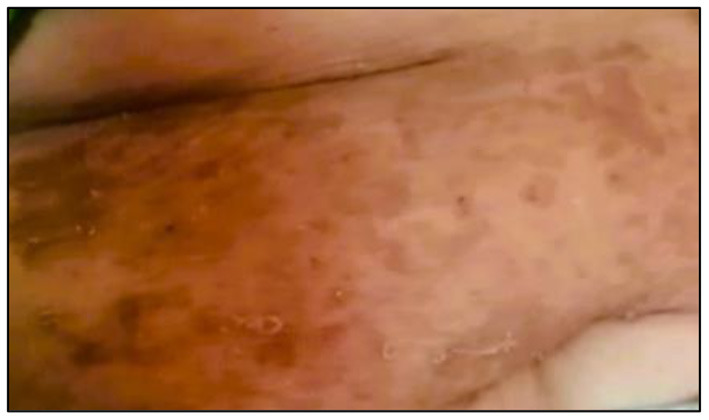
Hyperpigmentation on the respondent's stomach.

**Figure 5:**
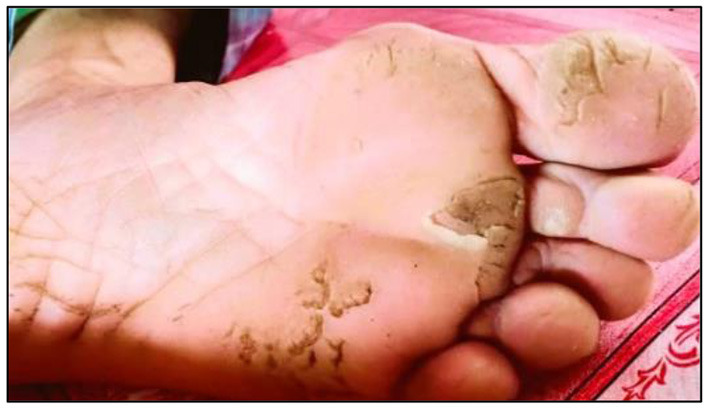
Hyperkeratosis on respondent's foot.

**Figure 6:**
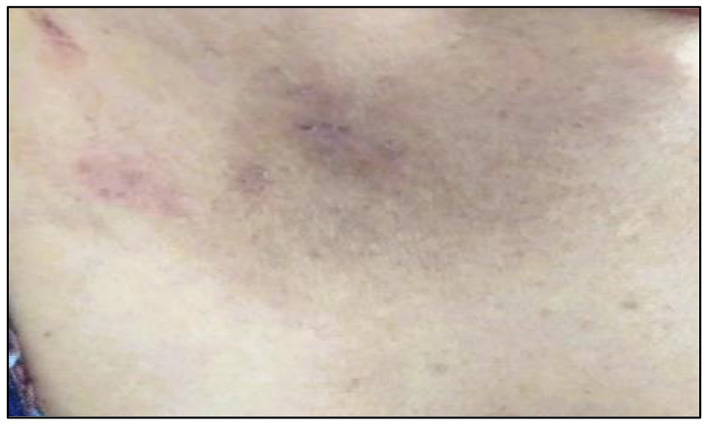
Hypermelanosis on the respondent's abdomen.

Out of the total number of respondents, 554 respondents had safe levels of hair arsenic (<1 μg/g), while 85 respondents had unsafe levels of hair arsenic (≥1 μg/g). Three arsenicosis signs were identified during the survey: hyperkeratosis, hyperpigmentation, and hypomelanosis (Figures 4–6). The results showed that the respondents with unsafe levels of arsenic demonstrated a higher prevalence of hyperkeratosis (10.6%), hyperpigmentation (9.4%), and hypomelanosis (4.7%) than those with low levels of hair arsenic. Hyperkeratosis prevalence showed a significant difference between respondents with high hair arsenic levels and those with low levels. A similar trend was also observed for study villages where the exposed village (Village AG) showed a significantly higher incidence of arsenicosis hyperkeratosis (8.3%), hyperpigmentation (9.7%), and hypomelanosis (4.3%) than those in Village P (Table 3). A confirmed diagnosis was defined when at least one sign of arsenicosis presence and hair arsenic equal to or >1 μg/g (21). Using these criteria, 18 respondents met the requirements, yielding a prevalence rate of 21.2%.

**Table 3:** Prevalence of signs of arsenicosis by the level of arsenic in hair and study village.

**Sign of arsenicosis**	**Level of arsenic in hair**				***P*** **-value**	**CI (95%)**	**Study village**				***P*** **-value**	**CI (95%)**
	<**1** μ**g/g** **(*****n*** = **554)**		≥**1** μ**g/g** **(*****n*** = **85)**				**Village AG** **(*****n*** = **324)**		**Village P** **(*****n*** = **315)**			
	***n***	**%**	***n***	**%**			***n***	**%**	***n***	**%**		
Hyperkeratosis	24	4.3	9	10.6	0.015*	1.17, 5.83	27	8.3	6	1.9	<0.001^*^	0.09, 0.55
Hypomelanosis	11	2.0	4	4.7	0.123	0.13, 1.32	14	4.3	1	0.3	0.001^*^	0.10, 0.55
Hyperpigmentation	31	5.6	8	9.4	0.171	0.25, 1.29	32	9.7	7	2.2	<0.001^*^	0.10, 0.50

* Significant at alpha 0.05.

The three most common symptoms reported by respondents were numbness of fingertips, palms, and soles, frequent headaches, and tingling sensation. All these symptoms, with the addition of forgetfulness and frequent nausea, were significantly higher for respondents with elevated hair arsenic levels (≥1 μg/g) than those with lower arsenic levels. When the symptoms were compared between the two villages, the prevalence of having symptoms of arsenicosis was greater in Village AG compared to the control, Village P. The symptoms of frequent stomachache, headache, forgetfulness, tingling sensation, and numbness of fingertips, palms, and soles were significantly higher in Village AG compared to Village P (Table 4).

**Table 4:** Prevalence of symptoms of arsenicosis by hair arsenic level and study village.

**Symptoms of arsenicosis**	**Hair arsenic level**				***P*** **-value**	**CI (95%)**	**Study village**				***P*** **-value**	**CI (95%)**
	<**1** μ**g/g (*****n*** = **554)**		≥**1** μ**g/g (*****n*** = **85)**				**Village AG (*****n*** = **324)**		**Village P (*****n*** = **315)**			
	***n***	**%**	***n***	**%**			***n***	**%**	***n***	**%**		
Difficulty in breathing	5	0.9	2	2.4	0.232	0.07, 1.98	5	1.5	2	0.6	0.270	0.08, 2.11
Coughing blood	1	0.2	1	1.2	0.126	0.01, 2.45	2	0.6	0	0	0.163	0.99, 1.02
Recurrent chesty cough	4	0.7	2	2.4	0.147	0.05, 1.67	5	1.5	1	0.3	0.108	0.02, 1.75
Frequent Stomachache	18	3.2	6	7.1	0.085	0.17, 1.15	22	6.8	2	0.6	< 0.001^*^	0.02, 0.38
Frequent nausea	4	0.7	3	3.5	0.021^*^	0.04, 0.90	6	1.9	1	0.3	0.062	0.02, 1.41
Frequent vomiting	2	0.4	1	1.2	0.306	0.03, 3.39	2	0.6	1	0.3	0.579	0.05, 5.68
Forgetfulness	13	2.3	7	8.2	0.004^*^	0.10, 0.70	16	4.9	4	1.3	0.008^*^	0.08, 0.75
Hallucination	2	0.4	0	0	0.579	0.99, 1.00	2	0.6	0	0	0.163	0.99, 1.02
Convulsion	2	0.4	0	0	0.579	0.99, 1.00	0	0	2	0.6	0.151	0.99, 1.00
Slow speech and movement	6	1.1	1	1.2	0.939	0.11, 7.74	4	1.2	3	1.0	0.732	0.17, 3.47
Frequent headache	54	9.7	15	17.6	0.029^*^	0.27, 0.94	55	17.0	14	4.4	< 0.001^*^	0.12, 0.42
Tingling sensation	43	7.8	14	16.5	0.009^*^	0.22, 0.82	43	13.3	14	4.4	< 0.001^*^	0.16, 0.57
Numbness fingertips,palm, soles	65	11.7	19	22.4	0.007^*^	0.26, 0.82	62	19.1	22	7.0	< 0.001^*^	0.19, 0.53
Weakness upper limb	19	3.4	5	5.9	0.268	0.21, 1.56	16	4.9	8	2.5	0.111	0.21, 1.19
Weakness lower limb	22	4.0	7	8.2	0.079	0.19, 1.11	14	4.3	15	4.8	0.789	0.53, 2.33
Leg cramps	8	1.4	1	1.2	0.845	0.15, 9.97	4	1.2	5	1.6	0.705	0.34, 4.85
Loss of weight	11	2.0	1	1.2	0.609	0.22, 13.35	8	2.5	4	1.3	0.264	0.15, 1.70
Loss of appetite	13	2.3	0	0	0.154	0.96, 0.99	8	2.5	5	1.6	0.430	0.21, 1.97

* Significant at alpha 0.05.

### Risk factors associated with hair arsenic levels of more than 1 ug/g

The result demonstrated that the risk factors of age, female gender, living in Village AG and smoking cigarettes were significantly associated with high hair arsenic levels in this study. The highest risk was living in Village AG, with an adjusted odd ratio (AOR) of 33.03. An increment of 1 year in age increased the risk of elevated hair arsenic levels by 2%. Females in this study had a 6.5-fold risk of having elevated hair arsenic compared to males and smoking also increased the risk of elevated hair arsenic by 4-fold compared to non-smokers (Table 5).

**Table 5:** Logistic regression analysis to determine factors associated with hair arsenic levels of more than 1 ug/g.

**Factor**	**Crude OR (95% CI)**	***P*** **-value**	**Adjusted OR^α^ (95% CI)**	***P*** **-value**
Age	1.02 (1.00, 1.03)	0.039^*^	1.02 (1.00, 1.04)	0.016^*^
**Gender**				
Male	1		1	
Female	1.7 (0.99, 2.82)	0.054	6.49 (2.18, 19.27)	0.001^*^
**Duration of stay**				
<10 years	1		1	
10 years and more	2.32 (1.29, 4.17)	0.005^*^	1.43 (0.74, 2.75)	0.288
**Study village**				
Village P	1		1	
Village AG	35.24 (11.0, 112.89)	<0.001^*^	33.03(9.95, 109.63)	<0.001^*^
**Smoking**				
No	1		1	
Yes	1.39 (0.79, 2.44)	0.26	4.26 (1.36, 13.34)	0.013^*^

* Significant at alpha 0.05.

α Enter method multiple logistic regression.

Multicollinearity and interaction terms were checked and not found.

Hosmer-Lemeshow test p = 0.764, classification table (overall correctly classified percentage = 86.7%) were applied to check the model fitness.

## Discussion

In this study, we found that arsenic concentration in Village AG drinking water was higher than the guidelines for drinking water by WHO and NDWQS (6, 22). However, the arsenic concentration in Village AG was not as high as in other countries such as Cambodia and Bangladesh. For example, Sthiannopkao et al. (23) and Gault et al. (24) reported that the arsenic concentration in Kandal, Cambodia's drinking water ranges from 5–543 to 0.21–943 μg/L, respectively. Meanwhile, in Bangladesh, the British Geological Survey in 1998 shown out of 2022 water samples collected, 35% had arsenic concentrations of more than 50 μg/L, and 8.4% were above 300 μg/L (25). The differences between our study and Bangladesh are probably due to different drinking water sources. In Village AG, the source of drinking water is mainly from the river; meanwhile, in those countries, their primary water sources are groundwater, that believed to have rich-arsenic content. Furthermore, arsenic contamination of the drinking water in Village AG is also believed to be a possibility due to seasonal variations. The Village AG drinking water collections were conducted in September and October during the rainy seasons. This finding is comparable with studies by Egbinola and Amanambu (26) and Savarimuthu et al. (27) showed that high arsenic concentrations were found during the early monsoon season, proving that seasonal disparities exist in contaminant concentration.

Hair is used as the biomarker in this present study to determine human exposure to inorganic arsenic. Compared to the other biomarkers such as urine and blood serum, hair had more advantages as it records chronic exposure to arsenic which occurs a few months before collection (24). Moreover, it is more convenient for collecting, storing, and transporting samples. Based on the findings of elevated arsenic in drinking water and hair of respondents in Village AG, it is highly suggestive that there has been chronic arsenic exposure among the residents as a level of more than 1.0 μg/g in hair usually indicates chronic intoxication (9, 28). Furthermore, many other studies reported that those exposed to prolonged environmental arsenic had a high arsenic concentration in their hair. For example, in the studies in Kandal Province, Cambodia, by Phan et al. (19), Sthiannopkao et al. (23) and Sampson et al. (29), the arsenic in hair ranged from 0.271–30.09, 0.06–30, and 0.05–13.94 μg/g respectively. However, using hair as a biomarker had limitations as the arsenic in hair could be influenced by pathways other than drinking water, such as the consumption of rice and vegetables that are high in arsenic and exposure to exogenous arsenic during bathing.

This study demonstrated that residents with high arsenic levels in the water had a higher incidence of arsenicosis. The result was equivalent to the study conducted in China (38), where villages with poor water supplies and high arsenic in water yielded 13.48–19.67% prevalence rate of arsenicosis. This finding proves that the arsenic level in water is one of the main factors contributing to the incidence of arsenicosis in the community. In this study, the most frequently found sign was hyperkeratosis among the respondents with hair arsenic levels of more than 1 ug/g. This result corresponds to studies in Bangladesh and Iran, where hyperkeratosis was reported to be more prevalent than other signs (30, 31). However, the study by Hashim et al. (21) reported that hypomelanosis was more prominent in Kandal, Cambodia. The differences between our findings and the previous study are probably due to the length of exposure and genetic and nutritional differences in the study area (31). Eighteen respondents from Village AG had their hair arsenic level equal to or more than 1 μg/g. They showed at least one sign of arsenicosis, which fulfilled the criteria of confirmed cases of arsenicosis. These respondents will further be evaluated by a dermatologist and screened for cancer, as chronic arsenicosis could lead to skin, bladder, and kidney cancer.

Arsenicosis symptoms vary, and most of them are the leading cause of morbidity and mortality associated with this disease. There are no specific symptoms of arsenicosis. Therefore, it could be confused with another disease which had similar symptoms. In this study, the prevalence of symptoms such as frequent nausea, forgetfulness, frequent headache, tingling sensation, and numbness of fingertips, palms, and soles was significantly higher for respondents having hair arsenic levels of 1 ug/g and more. Previous studies supported this by stating that symptoms such as nausea could manifest due to chronic arsenic exposure in the gastrointestinal system (9, 32). A systematic review by Brinkel et al. (33) revealed that various studies confirmed that exposure to arsenic will modify the physiology of the brain and might lead to developmental disabilities and intellectual disability; this is because arsenic has the ability to cross the blood-brain barrier and affect the central nervous system. (33). A Taiwan study shows that adolescents' neurobehavioral function may be affected by childhood exposure to arsenic in drinking water (34). In Cambodia, children with high hair arsenic levels experienced a 1.57–4.67 times greater risk of having lower neurobehavioral test scores than those with low hair arsenic levels after adjusting for hair lead, manganese and cadmium (35).

In our study, females demonstrated higher arsenic concentration in hair than males. Most females in this study are housewives; thus, there could be a probability that the exposure to the contaminated drinking water is more remarkable as they used the contaminated water for cooking, drinking, bathing and other. Compared to males who work outside the village, they probably get their drinking water from other sources that are not contaminated with arsenic. This finding differs from the previous study, which reported that males are prone to have high hair arsenic compared to females as they work as a labor and drink more water containing arsenic (3, 36). We also found that smoking can increase the risk of having a high arsenic concentration in hair. A study in Austria which looked into the influence of tobacco on heavy metals in the hair shows that in smokers, the geometric mean of hair arsenic was 0.081 ug/g higher than non-smokers, 0.065 ug/g, thus supporting the finding in this study (37). According to WHO, the tobacco plant can take up natural inorganic arsenic, which is naturally present in soils, thus indirectly can expose smokers to the carcinogenic element. Therefore, smokers with high hair arsenic could probably be exposed to either inhaling tobacco or ingesting water contaminated with arsenic. However, both synergic effects are harmful as they increase lung cancer risk (37).

## Conclusion

This study proved that arsenic was present in residents daily drinking water distributed from AG WTP. The study also found that people who live in an area exposed to high levels of As yielding high arsenicosis incidence. Although the arsenic contamination in Village AG is not severe as reported by other countries, the confirmed case of arsenicosis shows an alarming indication of chronic exposure, which could jeopardize public health in future. However, the finding in this study is only the tip of the iceberg of the arsenic pollution problem. Therefore, the policy-making bodies should find the primary source of arsenic pollution in the area to help eradicate the contamination.

## Further improvement

Evidence from this study has been reported to the responsible authorities for further mitigation action. Subsequently, the authorities took immediate measures by shutting down the affected water treatment plant. Furthermore, they installed a new pipeline from another uncontaminated water treatment plant to provide arsenic-safe drinking water for residents in Village AG. However, long-term monitoring should be conducted to guarantee the safety of drinking water and track the progressive effectiveness of alternative arsenic-safe water supplies.

## Data availability statement

The original contributions presented in the study are included in the article/supplementary material, further inquiries can be directed to the corresponding author.

## Ethics statement

The studies involving human participants were reviewed and approved by Medical Research and Ethics Committee, Ministry of Health Malaysia. The patients/participants provided their written informed consent to participate in this study.

## Author contributions

NR contributed to the data collection, analysis, and manuscript writing. HA contributed to the funding acquisition, study conception, and data collection. WW contributed to study design and manuscript writing. ZH and JH contributed to data interpretation. RS contributed to the funding acquisition, study conception, and design. All authors reviewed the manuscript.
